# The Adjuvant Effect of Hyperbaric Oxygenation for *Loxosceles rufescens* Bite: A Case Series

**DOI:** 10.3390/metabo15070470

**Published:** 2025-07-10

**Authors:** Simona Mrakic-Sposta, Alessandra Vezzoli, Carmela Graci, Maristella Gussoni, Attilio Cimmino, Cinzia Dellanoce, Enrico Maria Camporesi, Giovanni Sesana, Gerardo Bosco

**Affiliations:** 1 Institute of Clinical Physiology, National Research Council (IFC-CNR), Piazza dell’Ospedale Maggiore, 3, 20162 Milan, Italy; maristella.gussoni@unimi.it (M.G.); cinziacarla.dellanoce@cnr.it (C.D.); 2 ASST Grande Ospedale Metropolitano Niguarda, Piazza dell’Ospedale Maggiore, 3, 20162 Milan, Italy; graci.carmela@gmail.com (C.G.); attiliocimmino@ospedaleniguarda.it (A.C.); giovanni.sesana@ospedaleniguarda.it (G.S.); 3 Department of Biomedical Sciences, University of Padova, 35131 Padova, Italy; ecampore@usf.edu (E.M.C.); gerardo.bosco@unipd.it (G.B.)

**Keywords:** brown spider, spider violin, oxidative stress, inflammation, hyperoxia, HBOT

## Abstract

Background. The venom of *Loxosceles* *rufescens* (L.r.), also known as the violin and/or brown spider, contains a wide variety of proteins and can induce a complex, intense, and uncontrolled inflammatory response, hemolysis, thrombocytopenia, dermo-necrosis, and renal failure. Studies have postulated the efficacy of hyperbaric oxygen therapy (HBOT) for *Loxosceles* bites. However, data describing the use and beneficial effects of HBO are, to date, relatively scarce. Only a few cases of *Loxosceles* bites in Northern Italy have been documented, and there is no laboratory test available for the diagnosis. Objectives. We present seven cases (aged 54.5 ± 4.2 years) of patients who presented to the emergency room (E.R.) of Niguarda Hospital in Milan from March to October 2022. Methods. Blood and urine samples were collected and biomarkers of oxidative stress (OxS) (reactive oxygen species (ROS), total antioxidant capacity (TAC), lipid peroxidation (8iso-PFG2α), DNA damage (8-OH-dG)), inflammation (IL-6, IL-1β, TNF-α, sICAM1), and renal function (creatinine, neopterin, uric acid) before (T0), during (T1, T2), and after (1–2 wk T3–T4; 1 month T5) the HBOT treatment (US Navy Treatment Table 15 protocol) were studied. Results. At T0, patients showed a significant unbalance of OxS; high levels of ROS, 8-isoPGF2α, and inflammatory status (IL-6, TNF-α; sICAM); and a low level of antioxidant capacity. At the end of HBOT (T2), a significant reduction in Oxy-inflammation levels over time—8-iso −26%, 8-OH-dG −9%, IL-6 −71%, IL-1bβ −12%, TNF-α −13%, and sICAM1 −17%—associated with clinical improvement was shown. Conclusions. These reductions, along with those in renal function markers, mirrored the observed improvement in the evolution of the skin lesion and the patients’ self-reported general wellness and pain. In conclusion, HBOT should be considered a valuable therapeutic tool after L.r. bites.

## 1. Introduction

*Loxosceles rufescens* (L.r.), known as the violin spider or fiddle-back spider or brown spider, is a small spider (body length of about 9 mm, paw length of 4–5 cm), of brown color, with a violin-shaped pattern on their cephalothorax, and characterized by six eyes. Originating in the Mediterranean region, particularly in southern Europe (i.e., Spain, Italy, and France), North Africa, and Iran, it now also inhabits the USA, China, Japan, Korea, India, Australia, Thailand, Hawaii, and South Africa [1,2,3]. Spider bites are challenging to confirm but may be clinically diagnosed by considering the geographic location, seasonality, and clinical characteristics [3,4,5]. Loxocelism is characterized by the appearance of a local, itchy, and progressively painful injury [6,7]. *Loxosceles* bites can cause necrotic ulcerations of various sizes and dimensions, starting as an erythematous macule and a central papule. Then, the process progresses to the necrotic phase, characterized by a purplish plaque, the onset of which occurs in the first 24–48 h post-bite. The lesion typically evolves into a central blister, surrounded by dusky macules and a ring of blanched skin, showing an asymmetrical erythematous border (known as the “halo effect”). Soft tissue necrosis has been documented following envenomation by spiders (L.r.), vipers, and even traumatic dog bites, due to venom-induced cytotoxicity or vascular compromise [8,9,10].

Necrosis is usually evident after 72 h [11,12,13]. The venom of L.r. contains a wide variety of proteins and can induce a complex, intense, and uncontrolled inflammatory response, accompanied by hemolysis, thrombocytopenia, dermonecrosis, and renal failure [13,14,15,16,17]. Additionally, it contains phospholipases D, capable of hydrolyzing sphingomyelin, leading to muscle fiber lysis. Subsequently, liberated ceramides act as intermediaries regulating TNF-α and recruiting neutrophils [17]. These neutrophils, in turn, generate reactive oxygen species (ROS)—such as hydroxyl, superoxide, peroxidase, and myeloperoxidase—contributing to tissue cytotoxicity and proinflammatory cytokine production [18,19]. Few studies have reported on oxidative stress responses following vipers or scorpions envenomation [20], but, to our knowledge, no data exist regarding oxidative stress in humans following spider bites.

As a consequence of the bite and the resulting soft tissue necrosis, several studies have proposed the potential efficacy of hyperbaric oxygen therapy (HBOT) in the treatment of brown spider bites; however, data that describe the use and the beneficial effect of HBOT are, to date, relatively scarce [21,22,23]. Zanon et al. (2016) described the successful use of HBOT in a patient treated for snake envenomation (*Atrox albinus* rattlesnake) [24]. Additionally, Marmo et al. (2017) reported its efficacy in treating wounds infected by *Capnocytophaga canimorsus* in preventing complications such as cellulitis, septicemia, meningitis, and endocarditis, which are also frequently associated with this pathogen [25]. Only a few cases of a *Loxosceles rufescens* bite in Northern Italy have been documented [26,27]: only eight are described in the Italian literature.

This paper presents seven documented cases of cutaneous loxoscelism in the Lombardy region (northern Italy; Lombardy is located between the Alps Mountain range and tributaries of the river Po). The symptoms usually start as local itching, erythema with mild pain, rapidly evolving into a necrotic lesion, and disappearing in several weeks when the eschar detaches.

The aim of the present study was to evaluate the effects of HBOT on *Loxosceles rufescens* bites accompanied by acute oxidative and inflammatory status (oxy-inflammation). Biomarkers of oxidative stress (reactive oxygen species, lipid peroxidation, DNA oxidation, and antioxidant capacity), inflammation (interleukin-6 and 1beta, tumor necrosis factor-alfa, and soluble intercellular adhesion molecule-1), and renal function (creatinine, neopterin, and uric acid) before, during, and after the HBO treatment were assessed.

## 2. Materials and Methods

### 2.1. Patients

The cases here presented include the analysis of seven patients (5 males, 2 females; mean age 54.5 ± 4.2 yr) with spider bites, treated at the ASST Grande Ospedale Metropolitan Niguarda, Milan, Italy, between March and October 2022. Currently, there is no laboratory test available for the diagnosis of *L. rufescens* bites [28,29,30]; therefore, identification was confirmed by an entomologist. Diagnosis was obtained by patients’ history, documented evolution, and hematological analysis. After wound evaluation and collection of venous blood samples for standardized biochemical–hematological analyses, patients were provided with prophylactic treatment: amoxicillin/clavulanate (1 g/200 mg intravenously three times/day) and cetirizine (1 cp/10 mg) [31,32]. Bite injuries varied significantly among patients in terms of severity, the development of systemic symptoms, and the extent of secondary dermonecrotic wounds. The study protocol was approved by the Human Ethical Committee (HEC-DSB/04-19) of the Department of Biomedical Science of the University of Padova (Italy), and all subjects provided informed consent. The study was carried out according to the Declaration of Helsinki.

### 2.2. Blood and Urine Samples

**Figure 1:**
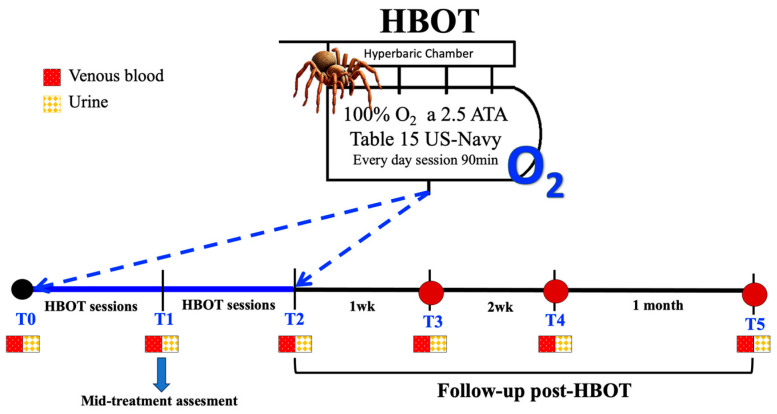
Experimental design of working protocol and timeline of blood and urine sample collection.

Blood and urine samples were collected on the 1st day of emergency room admissionn (T0), at the mid-point of HBO treatment (T1), at the end of HBO treatment (T2), and during recovery phase: at the 1st week (T3), 2nd week (T4), and 1 month (T5) post-HBOT (Figure 1). For each patient, approximately 5 mL of venous blood was drawn into EDTA and lithium heparin (LH) (Vacuette tube, Greiner bio-one, Kremsmünster, Austria). Blood samples were centrifugated for 10 min to separate plasma and red blood cells (RBC). Plasma samples were collected to determine levels of reactive oxygen species (ROS), antioxidant capacity (TAC), interleukins (IL-6 and IL-1β), tumor necrosis factor-alpha (TNFα), and soluble intercellular adhesion molecule-1 (sICAM-1). Urine samples were collected via voluntary voiding into a sterile container provided to the subjects and were used to determine lipid peroxidation (8iso-pGF2α), DNA damage (8-OH-dG), neopterin, creatinine, and uric acid concentrations. Multiple aliquots of plasma and urine were immediately frozen and stored at −80 °C.

### 2.3. HBOT Protocol

After a specialist medical examination, the patients were referred for hyperbaric oxygen therapy (HBOT) in the hospital, in accordance with recommendations from previous case reports [23,33,34,35,36,37]. Indeed, HBOT has been shown to neutralize the necrotizing components of *Loxosceles* venom and has been successfully used in the management of necrotic wounds. Patients were treated in a multiplace pressure chamber, receiving once-daily 90-minute sessions of HBOT with exposure to 100% oxygen at 2.5 ATA, following US Navy Treatment Table 15 protocol. Treatment duration ranged from a minimum of 8 to a maximum of 16 sessions, depending on the severity of the lesion caused by brown spider envenomation (Table 1). No experiences of adverse effects during hyperbaric therapy were reported.

**Table 1:** The characteristics, symptoms, and treatment of subjects. Ø = diameter; L = length; W = width; D = depth; min = minutes.

ID	Data Bite	Gender	Bite Location	Spider Bite Site	Bite Measure	Symptoms	HBOT
**1**	March 2022	M	Probably in the North Park in Milan	Left leg	15 L × 15 W × 3 D mm (Blister 30 × 20 mm)	Pain. Erythema, swelling, warmth. Fever 39 °C	n = 16 sessions 2.5 ATA 90 min Table 15
**2**	April 2022	F	Near Milan (north-west area)	Neck	70 L × 30 W mm primary lesion and 30 L × 10 W mm secondary lesion	Macular rash, diarrhea, headache, and general malaise. Fever 38 °C	n = 8 sessions 2.5 ATA 90 min Table 15
**3**	April 2022	M	Milan (north area)	Buttock	30 L × 20 W × 2 D mm	Vomit, swelling, warmth. Fever 38 °C	n = 9 sessions 2.5 ATA 90 min Table 15
**4**	May 2022	M	Milan (north area)	Left hip	20 L × 10 W mm lesion necrotic on the hip and 100 L × 10 W mm on the leg	Burning, swelling. Fever 38 °C	n = 8 sessions 2.5 ATA 90 min Table 15
**5**	July 2022	M	Milan (north-west area)	Left buttock and arm	150 L × 100 W mm primary lesion and 10 L × 7 W mm secondary lesion	Pain, swelling. Fever 38 °C.	n = 9 sessions 2.5 ATA 90 min Table 15
**6**	August 2022	F	Varese	Left shoulder	Ø80 mm × 50 W mm and 5 D mm (Blister)	Swelling, warmth, pus. Fever 38 °C.	n = 14 sessions 2.5 ATA 90 min Table 15
**7**	October 2022	F	Near Milan (north-east area)	Left ankle	Ø20 mm × 3 D mm (Blister)	With pain. Erythema, pus, swelling.	n = 16 sessions 2.5 ATA 90 min Table 15

### 2.4. Biomarker Measurements

#### 2.4.1. Biochemical and Hematologic Parameters

The evaluated biochemical and hematologic parameters were processed at Niguarda Hospital in the laboratory of clinical biochemistry: leukocites (WBC), erythrocytes (RBC), hemoglobin (Hb), hematocrit (HCT), mean corpuscular volume (MCV), mean corpuscular hemoglobin (MCH), mean corpuscular hemoglobin concentration (MCHC), and red cell distribution width (RDW); platelets (PLT), neuthrophils (%), lymphocytes (%), monocytes (MON%), eosinophils (%), basophiles (%), neuthrophils, lymphocytes, monocytes, eosinophils, and basophiles; Prothrombin Time (PT), International Normalized Ratio (INR), and Partial Thromboplastin P-Time (PTT); S-Alanine Aminotransferase, S-creatinine Kinase, S-creatinine, S-glucose, S-urea, S-total Ca, S-Na, S-K, A-alpha-Amylase, S-total Bilirubin, and S-Reactive Protein C (CRP).

#### 2.4.2. Oxidative Stress biomarkers

Reactive oxygen species signals were recorded by X-band Electron Paramagnetic Resonance (EPR; e-scan, Bruker, Berlin, Germany) at 37 °C (Noxigen Science Transfer & Diagnostics GmbH, Elzach, Germany). Spin-probe 1-hydroxy-3-methoxycarbonyl-2,2,5,5 tetramethyl-pyrrolidine-hydrochloride (CMH) was used. ROS were converted into absolute concentration values (μmol·min^−1^) by using the CP**•** (3-carboxy-2,2,5,5-tetramethyl-1-pyrrolidinyloxy) stable radical as an external reference. Spectra acquired were recorded and analyzed using Win EPR software (version 2.11), standardly supplied by Bruker. Details on the procedures have been previously reported by some of us [38,39,40,41].

The 6-hydroxy-2,5,7,8-tetramethylchroman-2-carboxylic acid (Trolox-) equivalent antioxidant capacity assay, a widely used kit-based commercial method (Cayman Chemical, Ann Arbor, MI, USA, Item No. 709001), was used as previously described [39,40,41]. Absorbance was measured at 750 nm using a spectrophotometer, and results were expressed in mM Trolox equivalents (mM).

Lipid peroxidation was assessed by immunoassay of 8-isoprostane (8-iso-PGF2α) concentration (Cayman Chemical, Ann Arbor, MI, USA, Item No. 516351) in urine as previously described [36,39]. Samples and standards were read in duplicate at a wavelength of 512 nm. Results were normalized to urinary creatinine.

DNA damage was quantified via immunoassay 8-OH-2-deoxyguanosine (8-OH-dG) EIA kit (Cayman Chemical, Ann Arbor, MI, USA) in urine. Absorbance was measured at 412 nm and normalized to creatinine values [42].

#### 2.4.3. Inflammatory Biomarkers

Interleukins IL-6 (Cayman Chemical, Ann Arbor, MI, USA, Item No. 501030), IL-1β (Cayman Chemical, Ann Arbor, MI, USA, Item No. 583311), and TNF-α (Fine Test, Wuhan, China, Cat. No. EH0302) plasmatic levels were measured using human interleukins ELISA kits, according to the manufacturers’ instructions [40,41,42,43].

For the soluble intercellular adhesion molecule-1 (sICAM-1), a member of the immunoglobulin supergene family, plasmatic levels were determined by an ELISA assay kit (BioVendor R&D, Brno, Czech Republic, Item No. RAF 102R) according to the manufacturer’s instructions.

All immuno-enzymatic determinations of oxidative stress were assessed using a microplate reader spectrophotometer (InfiniteM200, Tecan, Grodig, Austria). Determinations were in duplicate, and the inter-assay coefficient of variation was in the range indicated by the kits’ manufacturers.

#### 2.4.4. Creatinine, Neopterin, and Uric Acid Concentration

Urinary creatinine, neopterin, and uric acid concentrations were measured by isocratic high-pressure liquid chromatography (HPLC; Varian ProStar, Burladingen/Germany). The method was previously described [38,39]. The calibration curves were linear over the range of 0.125–1 μmol/L, 3.75–60 mmol/L, and 1.25–10 mmol/L for neopterin, uric acid, and creatinine levels, respectively.

### 2.5. Visual Analog Scales

A visual analog scale (VAS) with items including general wellness, rested/tired, calm/agitated, headache/no headache, nausea/no nausea, and local pain (specific pain in the localized area of the bite) [39,41,43] was used to measure subjective mood. A VAS was assessed at baseline (T0), mid-HBO treatment (T1), and end of HBOT (T2).

### 2.6. Statistical Analysis

Values are expressed as mean ± standard deviation of the mean (SD). Kolmogorov–Smirnov test was implemented to assess whether each variable followed a normal distribution, and descriptive statistics were calculated. To estimate the significance, one-way ANOVA with a Bonferroni post hoc test was applied. A *p* value < 0.05 was considered statistically significant. Change ∆% estimation (([post value − pre value]/pre value) × 100) was also reported in the text. Statistical analysis was performed using the software GraphPad Prism package for Mac (GraphPad Prism 10.4.2, GraphPad Software Inc., San Diego, CA, USA).

## 3. Results

### 3.1. Biochemical–Hematological Parameters

Our data showed four out of seven patients with high Leukocytes levels (57%); one patient showed low erythrocytes, hemoglobin, and hematocrit (14%); all patients showed an increase in neutrophils (100%); two patients’ showed low levels of lymphocytes (28.5%); and three patients showed high levels of monocytes (43%). Also, high INR values were found in two out of seven patients (28.5%), and, in one, high levels of Prothrombin Time were found (14%). Finally, three patients showed high levels of S-Alanine Aminotransferase (43%); one showed high levels of S-Creatine Kinase (14%); two showed high levels of S-Creatinine (28.5%); and one of S-total Bilirubin (14%); all showed high values of S-Reactive Protein C (100%) (Table 2).

### 3.2. Oxidative Stress

**Figure 2:**
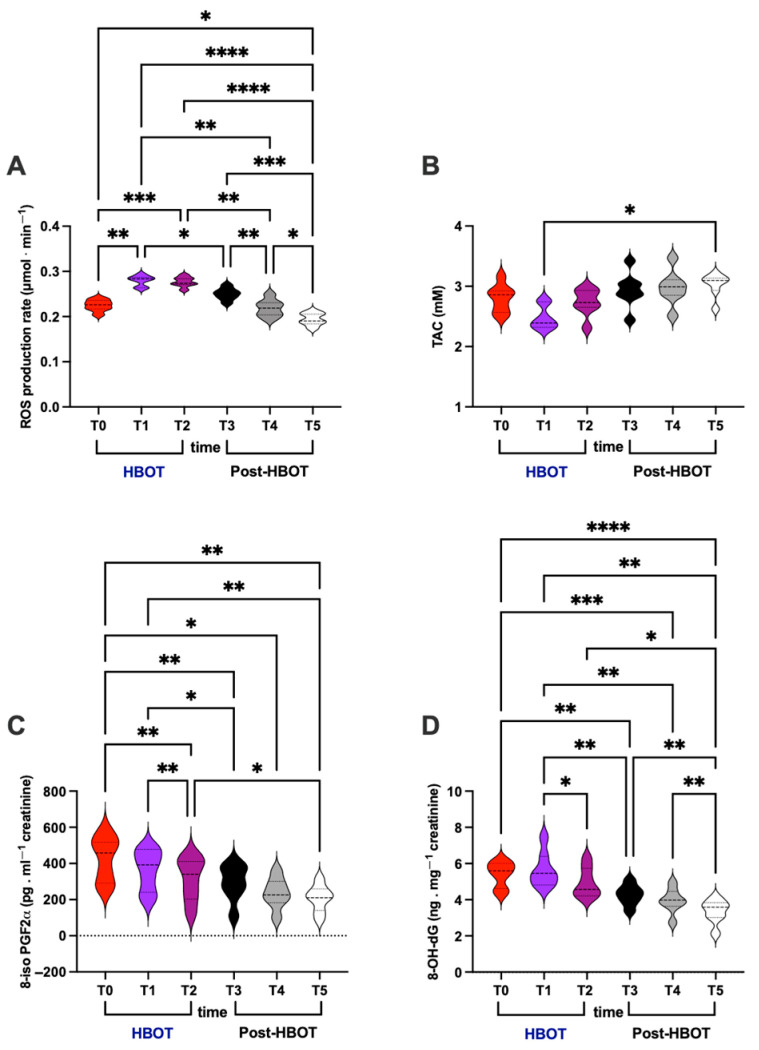
(**A**) Reactive oxygen species (ROS) production, (**B**) total antioxidant capacity (TAC) concentration assessed in plasma, and (**C**) lipid peroxidation and (**D**) DNA damage level detected in urine, at basal (T0), mid-HBOT treatment (T1), at the end of HBOT treatment (T2), and during recovery post-HBOT at 1st week (T3), 2nd week (T4), and 1 month (T5). Statistically significant difference comparisons are displayed as * *p* < 0.05; ** *p* < 0.01; *** *p* < 0.001; **** *p* < 0.0001.

ROS production rate, TAC in plasma, and oxidative damage biomarkers concentrations of lipid peroxidation (8-iso-PGF2α) and DNA damage (8-OH-dG) in urine were assessed. HBO treatment effects and recovery (post-HBOT at 1st and 2nd week and after 1 month) are displayed in Figure 2. The statistically significant differences, calculated from the collected data among different times, are herein reported.

In detail, significant differences were calculated as follows:

(i) ROS production rate (*μ*mol⋅min^−1^; Figure 2A)

HBOT: T0 vs. T1 and T2 (0.224 ± 0.012 vs. 0.279 ± 0.011 and 0.276 ± 0.011);

Post-HBOT: T3 vs. T4 and/vs. T5 (0.250 ± 0.013 vs. 0.218 ± 0.018 and/vs. 0.194 ± 0.013);

Overall: T1 vs. T3, and T4, and T5 (0.279 ± 0.011 vs. 0.250 ± 0.013 and 0.218 ± 0.018 and (0.194 ± 0.013), T2 vs. T4 and T5 (0.224 ± 0.012 vs. 0.218 ± 0.018 vs. 0.194 ± 0.013), and, above all, T0 vs. T5 (0.224 ± 0.012 vs. 0.194 ± 0.013).

(ii) TAC (mM; Figure 2B)

Overall: T1 vs. T5 (2.481 ± 0.196 vs. 3.023 ± 0.197).

(iii) 8-iso-PGF2α (pg⋅mg^−1^ creatinine; Figure 2C)

HBOT: T0 vs. T2 (426.9 ± 124.8 vs. 314.3 ± 121.3), T1 vs. T2 (362.2 ± 114.0 vs. 314.3 ± 121.3);

Overall: T0 vs. T3, and T4 (426.9 ± 124.8 vs. 293.1 ± 101.0 and 236.1 ± 84.43); T2 vs. T5 (314.3 ± 121.3 vs. 207.9 ± 74.15); T1 vs. T3, and T5 (362.2 ± 114.0 vs. 293.1 ± 101.0 vs. 207.9 ± 74.15); and, above all, T0 vs. T5 (426.9 ± 124.8 vs. 207.9 ± 74.15).

(iv) 8-OH-dG (ng⋅mg^−1^creatinine; Figure 2D)

HBOT: T1 vs. T2 (5.567 ± 0.968 vs. 4.942 ± 0.825);

Post-HBOT: T3 and T4 vs. T5 (4.364 ± 0.575 and 3.934 ± 0.686 vs. 3.352 ± 0.659);

Overall: T0 vs. T3, and T4 (5.403 ± 0.703 vs. 4.364 ± 0.575 and 3.934 ± 0.686); T1 and T2, and T3 vs. T5 (5.567 ± 0.968, and 4.942 ± 0.825 and 4.364 ± 0.575 vs. 3.352 ± 0.659); and, above all, T0 vs. T5 (5.403 ± 0.703 vs. 3.352 ± 0.659).

### 3.3. Inflammation

**Figure 3:**
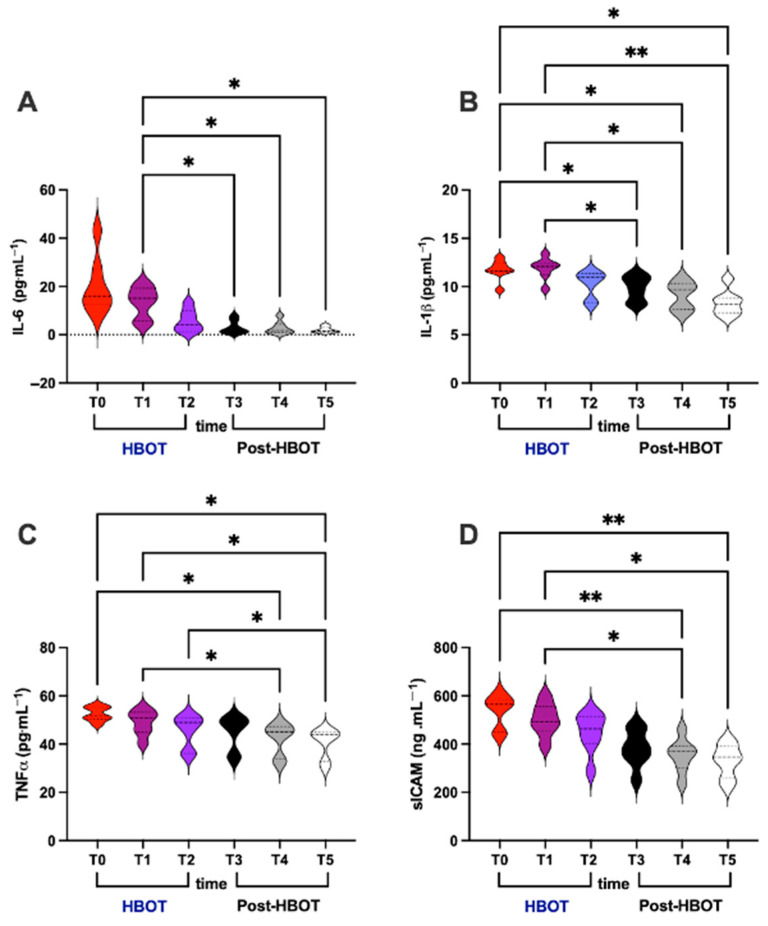
Cytokines (**A**) Il-6, (**B**) IL-1β, (**C**) TNF-α, and (**D**) sICAM levels assessed in plasma, at basal (T0), mid-HBOT treatment (T1), at the end of HBO treatment (T2), and during recovery post-HBOT at 1st week (T3), 2nd week (T4), and 1 month (T5). Statistically significant difference comparisons are displayed as * *p* < 0.05; ** *p* < 0.01.

Concerning inflammation status, IL-6, IL-1β, TNF-α, and sICAM levels were assessed in plasma. HBO treatment effect and recovery (post-HBOT at 1st and 2nd week and after 1 month) are displayed in Figure 3.

In detail, significant differences were calculated as follows:

(i) IL-6 (pg⋅mL^−1^; Figure 3A)

Overall: T1 vs. T3, and T4, and T5 (13.45 ± 6.44 vs. 3.08 ± 2.78 and 2.77 ± 2.63 and 3.01 ± 1.23).

(ii) IL-1β (pg⋅mL^−1^; Figure 3B)

Overall: T1 vs. T3, and T4 (11.87 ± 1.14 vs. 9.81 ± 1.31 and 9.06 ± 1.44); T2 vs. T3, and T4 and T5 (10.26 ± 1.38 vs. 9.81 ± 1.31 and 9.06 ± 1.44 and 8.28 ± 1.33); and, above all, T0 vs. T5 (11.70 ± 1.06 vs. 8.28 ± 1.33).

(iii) TNF-α (pg⋅mL^−1^; Figure 3C)

Overall: T0 vs. T4 (52.48 ± 2.98 vs. 42.33 ± 6.61); T1 vs. T4 and T5 (48.98 ± 5.22 vs. 42.33 ± 6.61 and 40.55 ± 6.21); T2 vs. T5 (45.51 ± 6.76 vs. 40.55 ± 6.21); and, above all, T0 vs. T5 (52.48 ± 2.98 vs. 40.55 ± 6.21).

(iv) sICAM (ng⋅mL^−1^; Figure 3D)

Overall: T0 vs. T4 (539.20 ± 68.25 vs. 353.50 ± 70.86); T1 vs. T4 and T5 (501.60 ± 68.58 vs. 353.50 ± 70.86 and 330.50 ± 68.08); and, above all, T0 vs. T5 (539.20 ± 68.25 vs. 330.50 ± 68.08).

### 3.4. Renal and Immunological Status

**Figure 4:**
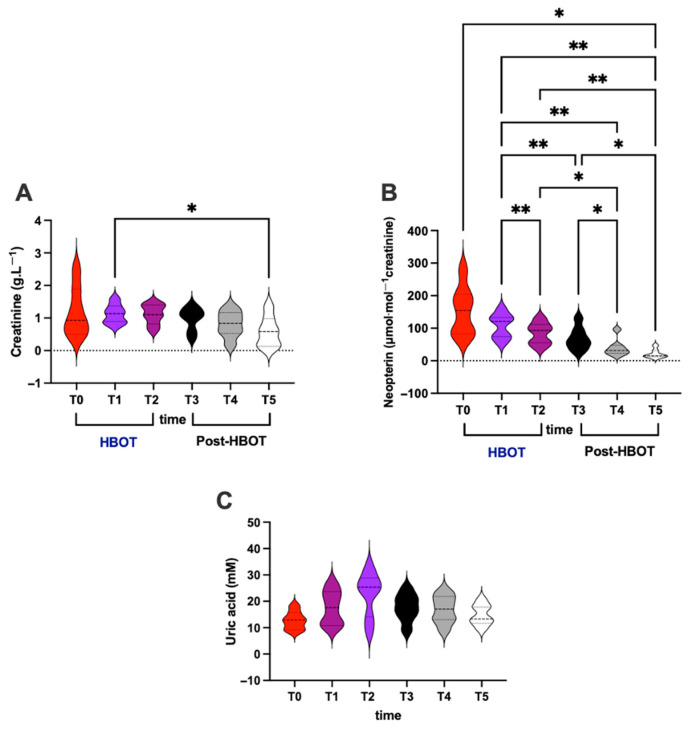
(**A**) Creatinine, (**B**) neopterin, and (**C**) uric acid concentration assessed in urine, at basal (T0), mid-HBOT treatment (T1), at the end of HBO treatment (T2), and during recovery post-HBOT at 1st week (T3), 2nd week (T4), and 1 month (T5). Statistically significant difference comparisons are displayed as * *p* < 0.05; ** *p* < 0.01.

Regarding renal and immunological status, creatinine and neopterin levels were assessed in urine. HBO treatment effect and recovery (post-HBOT at 1st and 2nd week and after 1 month) are displayed in Figure 4.

In detail, significant differences were calculated as follows:

(i) Creatinine (g⋅L^−1^; Figure 4A)

Overall: T1 vs. T5 (1.12 ± 0.29 vs. 0.58 ± 0.46).

(ii) Neopterin (*μ*mol⋅mol^−1^ creatinine; Figure 4B)

HBOT: T1 vs. T2 (107.70 ± 37.86 vs. 84.21 ± 33.23);

Post-HBOT: T3 vs. T4 and T5 (68.76 ± 35.46 vs. 39.93 ± 27.07 and 21.32 ± 13.35);

Overall: T1 vs. T3, and T4, and T5 (107.70 ± 37.86 vs. 68.76 ± 35.46 and 39.93 ± 27.07 and 21.32 ± 13.35), T2 vs. T4 and T5 (84.21 ± 33.23 vs. 39.93 ± 27.07 and 21.32 ± 13.35), and, above all, T0 vs. T5 (107.70 ± 37.86 vs. 21.32 ± 13.35).

No significant differences were found in uric acid concentration (Figure 4C).

### 3.5. Visual Analog Scale

**Figure 5:**
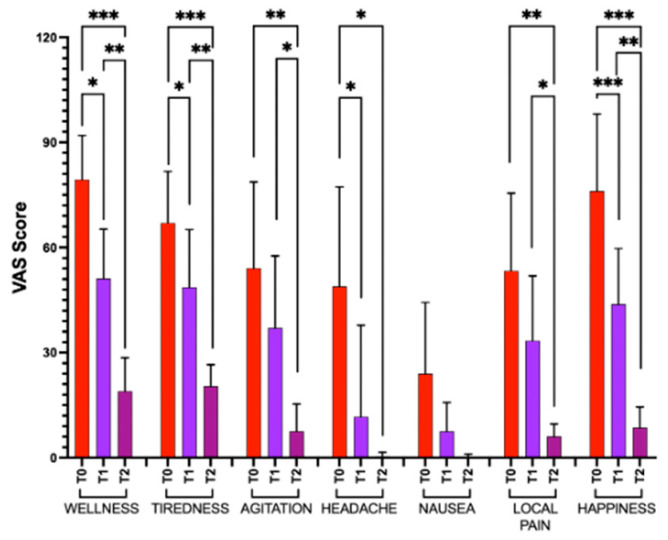
VAS scores for general wellness, tiredness, agitation, headaches, nausea, local pain, and happiness were assessed at the baseline (T0), mid-HBO treatment (T1), and at the end of HBOT (T2). Statistically significant difference comparisons are displayed as * *p* < 0.05; ** *p* < 0.01; *** *p* < 0.001.

Figure 5 shows reported significant changes in VAS item score at baseline (after spider bite; T0), mid-treatment HBOT (T1), and at the end of HBOT (T2). Particularly, we observed percentage changes at T2 with respect to the following: T0 in the general wellness: −76%, tiredness: −70%, agitation: −86%, headache: −99%, nausea: −99%, local pain: 88%, and happiness: −89%.

### 3.6. Clinical Images of Patients with a Loxosceles Rufescens Bite

**Figure 6:**
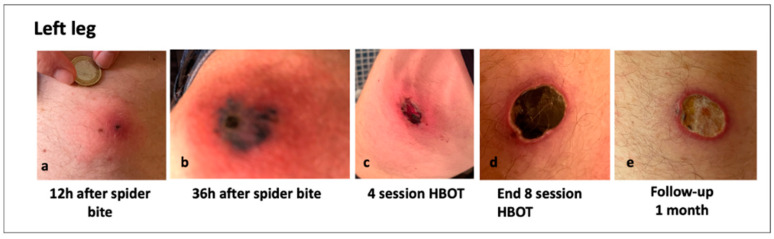
Time course of the site of envenomation from presentation to evolution in a male reporting a bite in left leg (thigh). The pictures show the evolution after bite: (**a**) about 12 h, and (**b**) about 36 h after bite. The bite points are clearly visible, with dark, necrotic center; (**c**) after 8 HBOT sessions, a significant improvement of local pain, general wellness, with no drainage and necrosis and appearance of fibrotic tissue; (**d**) at the end of 16 HBOT sessions, and in (**e**) the follow-up 1 month after. Complete wound healing with about 8 weeks after HBOT.

**Figure 7:**
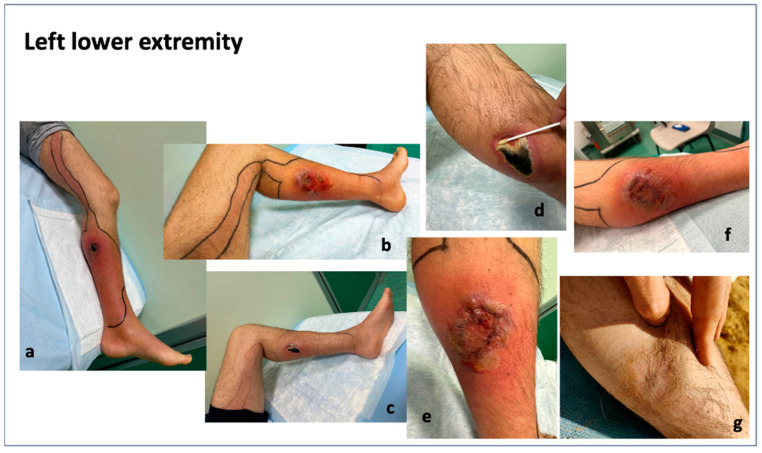
Time course of the site of envenomation in a male reporting a bite on his leg (calf). (**a**–**c**) Necrotic skin wound located in the lower third of the leg, associated with swelling, erythema, local pain, and warmth extended throughout the leg, associated with systemic symptoms (i.e., fever, high WBC, and PCR). Pictures show an inflammatory area with central flaccid blister. The necrotic superficial dermal tissue was debrided, the wound exhibited purulent material (**d**) and formation of the necrosis. After 14 HBOT sessions, the skin showed great improvement with disappearance of necrosis and formation of granulation tissue (**e**–**f**). Complete wound healing with scarring was observed 9 weeks after HBOT (**g**).

To further support the findings obtained from oxidative stress and inflammation markers, both before and after HBOT, in Figure 6 and Figure 7, photographic documentation of the spider bite lesions and their progression throughout the course of treatment is provided.

These pictures clearly illustrate the clinical improvements observed at the cutaneous level. Specifically, a marked reduction in erythematous areas is evident, accompanied by the gradual appearance of fibrotic tissue, indicative of ongoing tissue repair and healing.

## 4. Discussion

All patients showed significant improvement following the HBO therapy, as demonstrated in Figure 6 and Figure 7. The treatment of L. recluse spider bites aimed to minimize inflammation and tissue necrosis, preventing bacterial superinfection, alleviating pain and other complications such as pyoderma gangrenosum [10,44] and hemolytic anemia [45]. This is the first case series of seven patients evaluating the effects of HBOT on *Loxosceles* spider bites, inducing acute oxy-inflammation. The HBOT was started immediately, the day of E.R. admission after the bite, and all patients experienced significant improvement in wound healing, oxy-inflammation, culminating in complete resolution with satisfactory scarring. The local signs observed in spider bite victims include both local and systemic inflammation, with erythema, pruritus. Loxoscelism causes significant leucocytosis, consistent with previous studies [45]. Acute intravascular hemolysis has been described as a key mechanism of systemic loxoscelism [46]. In our cases, all patients showed high neutrophil counts and elevated C-Reactive Protein, indicating an inflammatory process, due to spider venom.

All patients presented at the emergency department with high levels of oxidative stress and inflammation. This aligns with the literature: Manzoni-de-Almeida and colleagues (2018) reported that the venom’s primary toxic component, sphingomyelinase D (SMaseD), induces an intense cascade of inflammatory reactions and oxidative responses, resulting in tissue damage and systemic complications [47,48]. Moreover, the authors highlight that in humans, SMaseD activates leukocytes, leading to ROS generation and the release of proinflammatory cytokines such as tumor necrosis factor-alpha (TNF-α). This activation is partially dependent on the complement system, suggesting a multifaceted immune response to the venom. Consistent with these findings, our data showed high levels of leukocytes as reported in Table 2, with elevated concentrations of ROS, IL-6, IL-1β, TNF-α, and sICAM1. Altogether, these activations suggest that the venom initiates a multilayered immunological and oxy-inflammatory cascade. Accordingly, a high level of neopterin was measured in our samples. Furthermore, as reported by de Souza and colleagues [49], *Loxosceles* venom can lead to complications such as hemolysis and acute kidney injury [50]. In our samples, after the bite, we found high levels of creatinine, suggesting kidney involvement.

As outlined in the introduction section, the HBO treatment, by enhancing oxygen delivery to hypoxic tissues, has been proposed to counteract the hypoxia and oxidative stress induced by *Loxosceles* venom. In the cases presented here, subjects underwent HBOT according to the US Navy Treatment Table 15 protocol. Post-treatment assessments (T2) showed significant reductions with respect to the baseline (T0) in oxidative (8-iso −26%; 8-OH-dG −9%) and inflammatory biomarkers (IL-6 −71%; IL-1β −12%; TNF-α −13%; sICAM-1 −17%), improvement in kidney function (creatinine −7%), and immunological responses (neopterin −45%), with clinical improvements in lesion healing (see Figure 4) and pain reduction (Figure 5; −88%). In summary, it is emphasized that HBOT positively influences the subjective mood state and contributes to tissue repair and wound healing.

These observations are supported by previous studies [23]. For instance, a case series reported successful healing of chronic necrotic ulcers caused by brown spider bites following HBOT (2ATA), even when initiated months after injury and where other therapies had failed, including topical dressings, antibiotics, and corticosteroids. Additionally, animal studies have shown that HBOT administered within 48 h of envenomation can reduce lesion size and severity. Specifically, HBOT has demonstrated specific beneficial mechanisms, including hyperoxia-induced inactivation of sphingomyelinase D [34,51,52]. At follow-up, one month after completing HBOT (duration varied by subject, see Table 1), all the analyzed parameters returned to normal values.

## 5. Limitations

One limitation of the study is certainly that no samples were collected at complete clinical recovery, which, as shown in Figure 6 and Figure 7, occurred at approximately 8–9 weeks, respectively. However, one month after the end of HBOT (duration varied by subject, see Table 1), all measured parameters had returned to normal. Additionally, no other data (e.g., subjective scale) were collected beyond this time point, limiting the ability to assess long-term changes.

## 6. Conclusions

In conclusion, the integration of HBOT into the treatment regimen for *L. rufescens* envenomation appears to offer substantial benefits. By enhancing oxygenation, HBOT modulates oxidative stress and inflammatory responses, potentially mitigating systemic complications. Moreover, HBOT supports wound healing and re-epithelialisation. Clinical observations suggest that HBOT accelerates granulation tissue formation and shortens wound closure time in necrotic spider bites. Based on these findings, emergency clinicians and wound care specialists should consider the early application of HBOT—ideally within the first 24–72 h after envenomation—as a supportive therapeutic strategy to improve patient outcomes, and reduce oxidative injury and inflammation. Given the absence of a specific laboratory diagnostic test for *Loxosceles* bites, the analyzed biomarkers could be considered valuable tools for assessing the efficacy of adjunctive treatments aimed at mitigating systemic complications such as renal impairment. Nonetheless, further clinical studies are necessary to establish evidence-based guidelines and assess long-term outcomes in necrotic wound healing related to *Loxosceles* envenomation.

## Figures and Tables

**Table 2:** Laboratory analysis data from patients with systemic loxoscelism at admission to Emergency Room. H: high; L: low. Values out of range are bolded.

Biochemical Hematological Parameters	1	2	3	4	5	6	7
**Leukocytes (WBC)** (10^9^/L) (range 4.5 to 11.0 × 10^9^/L)	**11.71 H**	9.53	**18.29 H**	8.83	**16.65 H**	**12.05 H**	9.95
**Erythrocytes (RBC)** (10^12^/µL) (range M: 4.7–6.1 10^12^/µL; F: 4.2–5.4 10^12^/µL)	4.83	4.81	5.21	4.73	4.66	4.50	**3.91 L**
**Hemoglobin (Hb)** (g/dL) (range M: 13.8–17.2 g/dL; F: 12.1–15.1 g/dL)	14	13.5	16.6	14.4	14.1	14.9	**11.9 L**
**Hematocrit (Hct)** (%) (range M: 40–54%; F: 36–48%)	42.8	41.8	48.7	42.5	42	43.6	**36.0 L**
**Corpuscular Volume** (fL) (range 80–100 fL)	88.6	86.9	93.5	89.9	90.1	96.9	92.1
**MCH** (pg) (range 27–33 pg)	29	28.1	31.9	30.4	30.3	**33.1 H**	30.4
**MCHC** (g/dL) (range 32–26 g/dL)	32.7	32.3	34.1	33.9	33.6	34.2	33.1
**RDW** (%) (range 11.5–14.5%)	13	14.4	13.2	12.6	13	14.4	13
**Platelets** (10^9^/L) (range 150–450 10^9^/L)	151	298	220	273	218	212	305
**Neutrophils** (%) (range 40–60%)	**76.1 H**	**65.3 H**	**78.5 H**	**82.2 H**	**88.3 H**	**70.3 H**	**71.2 H**
**Lymphocytes** (%) (range 20–40%)	**12.7 L**	24.3	**11.4 L**	**8.9 L**	**4.3 L**	**19.7 L**	21.3
**Monocytes** (%) (range 2–10%)	**10.9 H**	6.8	9.7	7.1	6.7	9.5	6.9
**Eosinophilis** (%) (range 0–6%)	0.0	2.8	0.1	1.7	0.5	0.3	0.4
**Basophiles** (%) (range 0.5–1%)	0.3	0.8	0.3	0.1	0.2	0.2	0.2
**Neutrophils** (10^9^/L) (range 2.0–7.0 × 10^9^/L)	**8.15 H**	**7.21 H**	**14.36 H**	**7.25 H**	**14.70 H**	**8.46 H**	**7.08 H**
**Lymphocytes** (10^9^/L) (range 1.5–3.5 × 10^9^/L)	1.36	2.32	2.8	**0.79 L**	**0.72 L**	2.37	2.12
**Monocytes** (10^9^/L) (range 0.2–0.8 × 10^9^/L)	**1.17 H**	0.65	**1.77 H**	0.63	**1.11 H**	1.15	0.69
**Eosinophilis** (10^9^/L) (range 0.04–0.4 × 10^9^/L)	0.00	0.27	0.02	0.15	0.09	0.04	0.04
**Basophiles** (10^9^/L) (range 0.01–0.1 × 10^9^/L)	0.0	0.1	0.1	0.0	0.0	0.0	0.0
**Prothrombin Time (PT)**							
**INR** (range 0.8–1.1)	1.08	0.97	1.13	1.08	**1.14 H**	**1.16 H**	**1.22 H**
**PT** sec (range 11–13.5 s)	13	12	14	13	13	13	14
**Partial Thromboplastin P-Time** (PTT) s (range 25–35 s)	27	28.7	**39.7 H**	27.7	25.1	29	33.5
**S-Alanine Aminotransferase** (U/L) (range M: 30 U/L; F: 19 U/L)	16	17	29	**58 H**	**50 H**	12	**188 H**
**S-Creatine Kinase** (U/L) (range 20–170 U/L)	140	51	114	52	67	**170 H**	85
**S-Creatinine** (mg/dL) (range M: 0.7–1.3 mg/dL; F: 0.6–1.1 mg/dL)	1.17	0.72	1.04	**1.26 H**	1.11	**1.1 H**	0.6
**S-Glucose** (mg/dL) (range 70–100 mg/dL)	**112 H**	86	**117 H**	**100 H**	**131 H**	**153 H**	94
**S-Urea** (mg/dL) (range 15–55 mg/dL)	27	29	41	39	45	26	25
**S-Total Ca** (mg/dL) (range 8.5–10.5 mg/dL)	9.5	9.5	10	9.5	9.4	9.6	9.4
**S-Na** (mmoli/L) (range 136–145 mmoli/L)	136	140	137	138	143	133	141
**S-K** (mmoli/L) (range 3.5–5.5 mmoli/L)	4.03	4.64	4.17	3.75	4.21	4.89	3.61
**S-Alpha-Amylase** (U/L) (range 30–110 U/L)	64	-	84	84	42	-	-
**S-Total Biliruin** (mg/dL) (range 0.2–1.0 mg/dL)	0.68	0.45	**5.87 H**	0.81	0.55	**1.07 H**	0.35
**S-Reactive Protein C (PRC)** (mg/dL) (range < 0.3 mg/dL)	**2.8 H**	**2.0 H**	**8.7 H**	**6.5 H**	**3.5 H**	**11.1 H**	**4.6 H**

## Data Availability

The original contributions presented in this study are included in the article. Further inquiries can be directed to the corresponding author(s).

## Abbreviations

The following abbreviations are used in this manuscript:

EPR	Electron Paramagnetic Resonance
ER	Emergency Room
Hb	Hemoglobin
Hct	Hematocrit
HBOT	Hyperbaric Oxygen Therapy
HPLC	High-pressure Liquid Chromatography
IL-1β	Interleukin-1 beta
IL-6	Interleukin-6
LR	*Loxosceles rufescens*
MCH	Mean Corpuscular Hemoglobin
MCHC	Mean Corpuscular Hemoglobin Concentration
OxS	Oxidative Stress
PCR	S-Reactive Protein C
PTT	Partial Thromboplastin P-Time
RBC	Red Blood Cell
RDW	Red Cell Distribution Width
ROS	Reactive Oxygen Species
sICAM1	Soluble Intercellular Adhesion Molecule-1
TAC	Total Antioxidant Capacity
TNF-α	Tumor Necrosis Factor-alpha
VAS	Visual Analog Scale
WBC	White Blood Cell
8-iso-PGF2α	Lipid Peroxidation
8-OH-dG	DNA Damage
